# Monitoring of the Environment at the Transplant Unit—Hemato-Oncology Clinic

**DOI:** 10.3390/ijerph110909480

**Published:** 2014-09-12

**Authors:** Ivanka Matoušková, Ondřej Holy

**Affiliations:** Department of Preventive Medicine, Faculty of Medicine and Dentistry, Palacký University Olomouc, Olomouc 775 15, Czech Republic; E-Mail: ivanka.matouskova@upol.cz

**Keywords:** stem cell transplantion, risks infection, cleanroom, monitoring microbial contamination of this environment

## Abstract

*Aims*: Aim of this study was to monitor the environment at the Transplant Unit—Hemato-Oncology Clinic, University Hospital Olomouc (Olomouc, Czech Republic) and identify risks for the patients. *Methods and Results*: Microorganisms were cultivated under standard aerobic conditions. Strains were biochemically identified using the BD Phoenix™ PID Panel (USA). *Legionella pneumophila* was identified by DNA sequencing. From the air, the most frequently isolated strains were coagulase-negative staphylococci (94.3%), *Micrococcus* spp. and *Bacillus* spp. No Gram-negative strains were isolated from the air. From the surfaces, the most frequently isolated Gram-positive strains were coagulase-negative staphylococci (67.4%), *Bacillus* spp., enterococci (5.5%), *Staphylococcus aureus* (2.3%) and *Micrococcus* spp. (1.7%). From the surfaces, the most frequently isolated Gram-negative strains were from genera *Pseudomonas* (28%), *Enterobacter* (28%), *E. coli* (6%), and *Klebsiella* spp. (5%). From the personnel, the most frequently isolated Gram-positive strains were coagulase-negative staphylococci (59.6%), *Bacillus* spp. (24.1%) and *Staphylococcus aureus* (9.8%). From the personnel, the most frequently isolated Gram-negative strains were *Enterobacter* spp. (61%), *Klebsiella oxytoca* (18%), and *E. coli* (11%). Microscopic filamentous fungi were isolated in 13 cases (2.71%). Isolated strains were *Aspergillus* spp. (4), *Trichoderma* spp. (2), *Penicillium* spp. (2), one case of the strains *Paecilomyces* spp., *Eurotium* spp., *Monilia* spp. *Conclusions*: The study found no significant deviations in the microbial contamination of the cleanroom air. The personnel entrance of the Transplant Unit represent a high risk area, an extreme value (7270 CFU/m^3^) was recorded. Regime measures are fully effective, no other deficiencies were found. *Significance and Impact of the Study*: This epidemiological study, which was held for the duration of one year at the Transplant Unit—Hemato-Oncology Clinic, University Hospital Olomouc. The study monitored microbial contamination of the cleanroom air, surfaces, water, colonization of the personnel by bacterial strains of epidemiological consequence.

## 1. Introduction

Stem cell transplantion is a life-saving treatment for many patients. In 2009, over 25,000 allogeneic and 30,000 autologous stem cell transplantations were performed worldwide. Infections cause 8% of patient deaths after autologous and 17–21% of them after allogeneic bone marrow transplantation [1].

Exogenous infections may be prevented in patients at the time of the highest risk with a special regime, using reverse isolation boxes with positive pressure—cleanrooms. In the introduction to HVAC (heating, ventilation, and air conditioning) Design Manual for Hospitals and Clinics, it is stated that temperature, relative humidity, and ventilation play an important role in the survival of microorganisms in cleanroom air.

This epidemiological study at the Transplant Unit—5C Department of the Hemato-Oncology Clinic, University Hospital Olomouc (Olomouc, Czech Republic) lasted one year (August 2010–July 2011). During the time of the study, a total of 96 patients were treated at the department, of whom 63 were transplant recipients. In 43 cases the transplantation was autologous and in the remaining cases the transplantation was allogeneic.

The Transplant Unit is a cleanroom of a specific class of air cleanliness (10,000) in accordance with FED-STD-209E [2]. The study monitored microbial contamination of the cleanroom air, surfaces, water, as well as colonization of the personnel by bacterial strains of epidemiological consequence. It consists of four patient isolation boxes and relevant sanitary facilities. Everything else, including filters in front of each isolation box are background facilities, which include the following premises: personnel locks adjacent to each room, a cleaning room, material room, kitchen, doctor's office, hallway and nurse break room.

The Transplant Unit is equipped with a HYD HKBCA 0150 air-conditioner (Nickel Prague, Prague, Czech Republic), which works exclusively with fresh air. The device conditions the air with a three-stage filtration system, heating, cooling, and steam humidification. Potable water is not suitable for patients. Therefore, potable water from the mains is treated using special technology. The water treatment equipment used for the Transplant Unit is supplied by Aqua Osmotic Tišnov (Aqua Osmotic Systems, Tišnov, Czech Republic). The water is pre-treated using the Aqua Osmotic 100K UV light system and reverse osmosis. The boilers heat the water to temperatures over 64 °C (prevention of the genus Legionella). In the sanitary facilities of the isolation boxes, the end of the shower hose and of the faucet are fitted with a terminal filter (Ionpure—Siemens, Hoffman Estates, IL, USA) with a filter membrane, 0.22 µm pore size (to prevent the entry of genus *Legionella*).

## 2. Materials and Methods

Procedures controlling microbial contamination of the cleanroom air, surfaces, items, and pre-treated water followed valid standards. Swabs detecting the bacterial colonization of healthcare workers were carried out in line with procedures described in literature [3]. Microorganisms were cultivated under standard aerobic conditions, using the culture media (Trios, Prague, Czech Republic) Thioglycollate broth (5 mL), Sabouraud broth (5 mL), Columbia blood agar, and Sabouraud glucose agar with chloramphenicol (chloramphenicol at a concentration of 50mg/L). Cultured bacterial strains were biochemically identified using the BD Phoenix™ PID Panel with a Phoenix Automated Microbiology System (Becton, Dickinson and Company, Sparks, MD, USA) at the Department of Microbiology, Faculty of Medicine and Dentistry, Palacký University Olomouc. Antibiotic sensitivity of selected bacterial strains was established using a micromethod of determining the minimal inhibitory concentrations (MIC) in a microtiter plate (Trios). *Legionella pneumophila* was identified by DNA sequencing—Sequence-Based Typing (SBT; EWGLI, www.ewgli.org), at the National Legionella Reference Laboratory, Ostrava Health Institute. Phenotypic identification of microscopic filamentous fungi was carried out at the Department of Microbiology, Faculty of Medicine and Dentistry, Palacký University Olomouc, and at the Culture Collection of Fungi, Department of Botany, Faculty of Science, Charles University (Prague, Czech Republic). Wilcoxon signed-rank test was used for statistical testing.

### 2.1. Assessment of Microbial Contamination of the Cleanroom Air

Microbial contamination of the cleanroom air at the Transplant Unit was monitored on a monthly basis. Twenty sampling sites were selected. In the patient isolation box, the air sampler was placed on the dining table, while in the sanitary facility and the personnel entrance it was stationed on the floor. At the other sampling locations, it was placed on an auxiliary trolley, about 110 cm above ground. A MAS-100 air sampler (Merck, Darmstadt, Germany) was used to actively suck 100 L of the air for 60 s. The cleanroom air was always sampled between 8 a.m. and 10 a.m., while the Transplant Unit was in operation. Columbia blood agar was used to determine the total number of microorganisms, while Sabouraud glucose agar with chloramphenicol (50 mg/L) in duplicate was used to identify microscopic filamentous fungi and yeast. A total of 720 samplings of cleanroom air were taken. The numbers of colonies cultured were converted with the nomogram and listed as cleanroom air CFU/m^3^ (colony forming units). The quantitative analysis was supplemented with a qualitative analysis. Measurements using the Testo 625 device (Testo AG, Lenzkirch, Germany) focused on the temperature and relative humidity of the air.

### 2.2. Assessment of the Microbial Contamination of HVAC Diffusers

Swabs of HVAC diffusers at the Transplant Unit rooms were always carried out following the sampling of the microbial contamination of the cleanroom air. The swabs were then cultured in duplicate in Sabouraud broth—TGPA (5 mL), and simultaneously in thioglycollate broth—Thi (5 mL).

### 2.3. Assessment of Microbial Contamination of the Surfaces

Microbial contamination of surfaces was monitored using the qualitative method. Swabs were carried out with a sterile synthetic swab applicator, always covering a 100 cm^2^ area. Samples of liquid soap and hand sanitizer were applied directly into thioglycolate broth. The Staphaurex^®^ rapid latex test kit (Remel Europe Ltd., Dartford, UK) was used to distinguish coagulase-negative staphylococci (CoNS) and *Staphylococcus aureus*.

### 2.4. The Sampling of Pre-Treated Water and Potable Water

As part of Legionella spp. surveillance, eight samples of pre-treated water (four showers, four faucets) were taken in March 2011 from the sanitary facilities of the patient isolation boxes. In the bathrooms of rooms no. 1 and 3, samples of water from the toilet tank were collected; the water is cold potable water supplied from the hospital’s mains and designated for flushing. The temperature of water samples ranged from 11.2 °C to 13.6 °C. In June 2011 a similar sampling was conducted, collecting water from the toilet tank in all four sanitary facilities. The temperature of water samples ranged from 10.8 °C to 13.1 °C. In addition, four swabs were taken from the inside wall of the toilet tank using a CLASSIQSwabsTM (COPAN, Brescia, Italy) sterile synthetic swab. A separate sampling of water was carried out in the shower of the personnel locks, which use hot water treated with chlorine dioxide from the hospital water system. The temperature of the sample was 14.4 °C. The isolates were identified by serotyping (panel *L*. *pneumophila sg* 1-16). Molecular typing of the *L. pneumophila* isolated strain was performed using DNA sequencing - Sequence-Based Typing (SBT).

### 2.5. Assessment of Personnel Colonisation with Bacterial Strains of Epidemiological Consequence

Swabs were taken from both nostrils/nasal mucosa, surface of the right hand and the scalp. The sterile swab soaked in sterile saline was used for the collection, and placed in a vial with 5 mL of thioglycolate broth.

## 3. Results

A total of 144 measurements of the temperature and relative humidity were taken throughout the study (Table 1).

**Table 1:** Temperature and relative humidity of the cleanroom air.

Microclimatic Parameters	Patient Isolation Box	Background Facilities
**Temperature (°C) **	24.0 ± 0.9 (21.8–26.3)	23.6 ± 0.7 (21.8–25.8)
**Rh (%) **	40.8 ± 14.4 (16.6–68.1)	40.0 ± 11.1 (19.3–57.4)

### 3.1. Determination of Microbial Contamination of the Cleanroom Air

**Figure 1:**
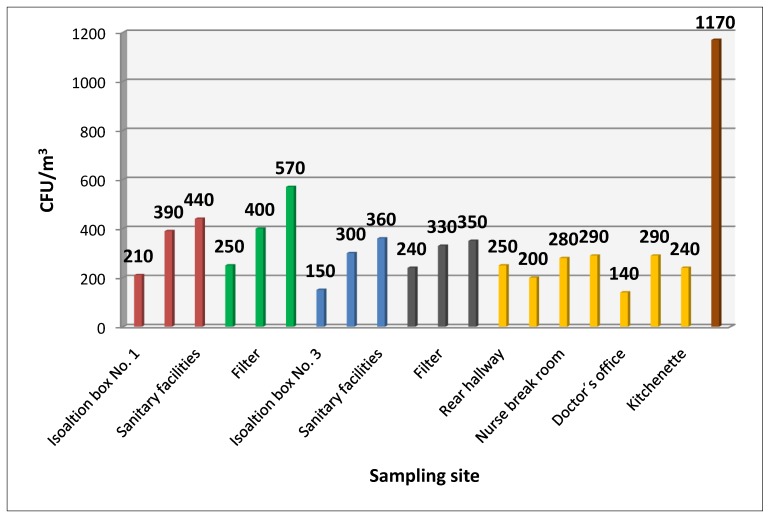
Average CFU/m^3^ at sampling sites.

**Figure 2:**
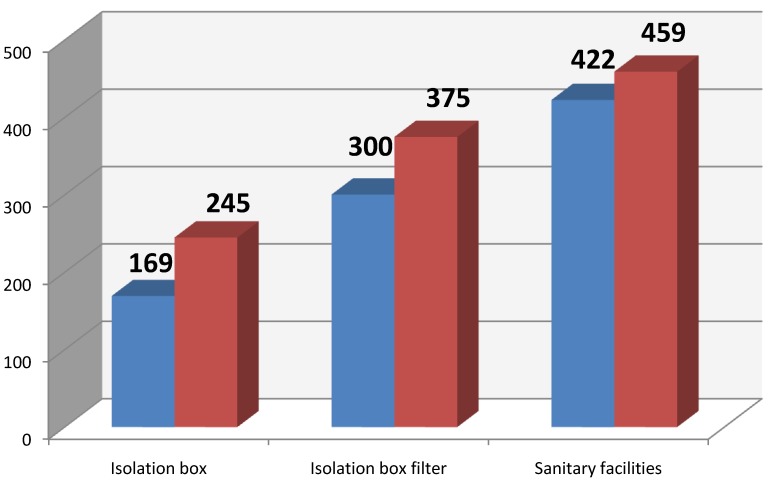
Average CFU/m^3^ (blue without patient, red with patient).

Figure 1 shows average values of cultured bacterial contamination at the sampling sites. Figure 2 shows the average microbial contamination of the cleanroom air of the patient isolation box, depending whether the patient was present or absent. Statistical evaluation did not show any significant difference concerning the presence/absence of the patient.

The bacterial strains isolated most frequently from the air were coagulase-negative staphylococci, which accounted for 94.3% of all detected bacterial strains. The second most frequently isolated strain was *Micrococcus* spp., followed by *Bacillus* spp.

### 3.2. Assessment of the Microbial Contamination of the Surfaces

The most commonly contaminated objects in the patient isolation box were the leatherette chair, followed by the table top and the inside of the refrigerator. Regarding the sanitary facilities, the highest incidence of bacterial contamination was found in the shower drain, water in the toilet bowl, and the basin drain. In the isolation box filter, the highest numbers of bacterial strains were detected on non-sterile gloves, scrubs and the stethoscope diaphragm. Throughout the study, the most frequent organisms isolated on the surfaces of the entire Transplant Unit were coagulase-negative staphylococci (67.4%), followed by *Bacillus* spp.—21.1% and, to a much lesser extent, by enterococci (5.5%), *Staphylococcus aureus* (2.3%) and the genus *Micrococcus* spp. (1.7%).

The most frequently isolated microorganisms from the surfaces of the entire Transplant Unit included representatives of the genera *Pseudomonas* (28%) and *Enterobacter* (28%), followed by *E. coli* (6%), and *Klebsiella* spp. (5%). “Others” include, by genera—*Acinetobacter*, *Kingella*, *Kluyvera*, *Ochrobactrum*, *Delftia*, *Morganella*, *Moraxella*, *Serratia* (in total these strains accounted for 20%). The most frequently isolated genus *Pseudomonas* were *Pseudomonas aeruginosa* (85%) and *Pseudomonas* putida (12%), with species *Pseudomonas* stutzeri recording the lowest incidence (3%). Concerning the genus *Enterobacter* spp., *Enterobacter cloacae* was isolated from surfaces in 77% and *Enterobacter aerogenes* in 23% of cases.

The highest number of microorganisms in the patient isolation box and the background facilities of the Transplant Unit were isolated from wash basin drains in the transplant unit’s background facilities and from shower drains, wash basin drains, and water in the toilet bowl in the sanitary facilities of the patient isolation box. Other surfaces showed only sporadic incidence of Gram-negative microorganisms. Other representatives that were isolated sporadically included e.g., *Listeria monocytogenes* from a cottage cheese container stored in the refrigerator of the patient isolation box. The most common enterococci isolated from the surfaces were *Enterococcus faecalis* (72%), followed by *Enterococcus faecium* (21%), and *Enterococcus caseliflavus* as the least frequent isolate (7%).

### 3.3. Personnel Colonisation with Bacterial Strains of Epidemiological Consequence

The findings of the epidemiological survey of personnel colonisation are divided into different perspectives:

(1) The most frequent Gram-positive organisms included coagulase-negative staphylococci (59.6%), followed by *Bacillus* spp. (24.1%) and *Staphylococcus aureus* (9.8%). The Gram-negative bacteria detected included representatives of the genus *Enterobacter* spp. (61%), *Klebsiella oxytoca* (18%), and *E. coli* (11%); the remaining strains were isolated sporadically (e.g., *Stenotrophomonas maltophilia*—4%). Of the *Enterobacter* genus, *Enterobacter aerogenes* was clearly prevalent, as its representatives formed a full 94% of all the *Enterobacter*s isolated from the clinical material of the personnel. The remaining 6% was *Enterobacter cloacae*.
(2) Bacterial strains divided by location (nostril/nasal mucosa, right hand, hair). In the case of Gram-positive strains, coagulase-negative staphylococci (57%) recorded the highest incidence, followed by *Staphylococcus aureus* (24%) and representatives of the genus *Bacillus* spp. In the case of Gram-negative bacteria, *Enterobacter aerogenes* (58%), *Klebsiella oxytoca* (26%), *E. coli* (16%) and *Enterobacter cloacae* (5%) were identified. Microorganisms obtained from the right hand were coagulase-negative staphylococci (55%), which recorded the highest incidence, followed by *Bacillus* spp. (39%). Gram-negative bacterial strains were represented by *Enterobacter aerogenes* (57 %), *Paracoccus yeeii* (14 %), *Pseudomonas mendocina* (14 %), and *Stenotrophomonas maltophila* (14 %). The Gram-positive strains that were most frequently identified in the hair of the personnel were coagulase-negative staphylococci (63%) and *Bacillus* spp. (27%). *Enterobacter aerogenes* was the microorganism that was most commonly isolated from the clinical material of the personnel; the most common site of the detection of Gram-negative strains was the nostril/nasal mucosa.
(3) Based on their profession, the personnel were divided to doctors, nurses, and employees of the ISS cleaning company. The division stems from the differences among the performed tasks that affect the contact with the patient (doctors, nurses—frequent contact; ISS employees—no contact) and the risk of cross transmission of infectious agents. The results share very similar characteristics; no major differences occur. In all the professions the most common Gram-positive strains are coagulase-negative staphyloccoci and the most common Gram-negative strains are the representatives of the *Enterobacter* genus. The incidence of the *Staphylococcus aureus* strain in the nasal mucosa was found to be higher in the employees of the ISS cleaning company than in nurses and doctors. The generic representation is relatively stable.

### 3.4. Identified Microscopic Filamentous Fungi in the Cleanroom Air

Microscopic filamentous fungi were cultivated in 13 cases (*i.e*., 2.71%): twice in samples from the sanitary facilities of the patient isolation box, while the other positive findings came from the facilities of the Transplant Unit. The most commonly cultivated genus was *Aspergillus* spp. (four instances—2x *Aspergillus terreus*, *Aspergillus sydowii* and *Aspergillus versicolor*), followed by two cases of *Trichoderma* spp., two cases of *Penicillium* spp., and one case of the strains *Paecilomyces* spp., *Eurotium* spp., and *Monilia* spp.

When the ground floor of the building was being renovated, no increased incidence of microscopic filamentous fungi spores was detected at the Transplant Unit. Throughout the entire study we found no representatives of microscopic filamentous fungi in the cleanroom air or on the surfaces of any of the four patient isolation boxes.

## 4. Discussion

Health care centres around the world have been struggling with hospital-acquired infections for over a century. These complex infections are characterized by multifactorial etiology. There are exogenous and endogenous risk factors for infectious complications. Exogenous factors include e.g., drugs, but also the environment of the health care centre where the patient is hospitalized. Endogenous factors depend on the recipient. Each environment of a health care centre has its own microbial colonization. The time interval between human activity and the measurement of microbial contamination is decisive. The literature draws attention to falsely high values of detected microorganisms in relation to cleaning or other activities [4]. During the study, no Gram-negative bacteria were identified in the cleanroom air of the Transplant Unit. This population was only identified on the surfaces and on the personnel of the Transplant Unit [5,6]. Before 2002, bacterial infections in neutropenic patients were caused by Gram-negative bacteria, but nowadays, it is Gram-positive strains. The most frequently reported etiological agents of infectious complications are coagulase-negative staphylococci and microscopic filamentous fungi. Their incidence has been on the rise since 2005 [7].

Bacterial strains isolated most frequently from the cleanroom air of the Transplant Unit were coagulase-negative staphylococci, which accounted for 94.3% of all detected bacterial strains. The second most frequently isolated strain was *Micrococcus* spp., followed by *Bacillus* spp. Bonetta observe that microorganisms that are cultured most frequently from cleanroom air include micrococci and staphylococci [8]. The principal source is human beings, followed by bioaerosols.

The measured microbial contamination of the cleanroom air in the personnel locks is closely linked to the overall high traffic at the Transplant Unit. It would be advisable to equip the locks with UV lights to reduce bacterial contamination of the cleanroom air. The role of UVC lamps in the decontamination of critical areas is highlighted in current literature [9].

Besides bacterial infections, immunocompromised patients very often contract fungal infections. Invasive fungal diseases (IFD) are an important cause of morbidity and mortality in immunocompromised patients recovering from allogeneic hematopoietic stem cell transplantation (HSCT). IFD prophylaxis includes two basic procedures: regime measures at health care units and pharmacological prophylaxis [10]. IFDs are prevented by hospitalizing the patient under a special accommodation regime in reverse isolation boxes with positive pressure—cleanrooms—with effective HVAC and built-in HEPA filters [11]. Some studies refer to Protected Environment (PE), designed primarily for patients recovering from allogeneic HSCT [12]. The technical parameters of isolation boxes with positive (negative) pressure, which are equipped with HEPA filters, are given by technical standards [2]. When the ground floor of the building was being renovated, no increased incidence of microscopic filamentous fungi spores was detected at the Transplant Unit. Throughout the entire study we found no representatives of microscopic filamentous fungi in the cleanroom air or on the surfaces of any of the four isolation boxes.

During the study, strains of *Trichoderma harzianum*, *Trichoderma citrinoviride*, and *Penicillium chrysogenum* were cultured from the cleanroom air of the doctor’s office. None of these strains were found at any other sampling location. The strains of *Trichoderma harzianum* and *Trichoderma citrinoviride* are regarded as an emerging cause of serious fungal infections in immunocompromised patients, especially haematology oncology patients. Infections caused by the *Penicillium chrysogenum* strain have been established in immunosuppressed patients and rarely in immunocompetent individuals [13].

The most commonly isolated microscopic filamentous fungus was the genus *Aspergillus* spp., which makes up to 40% of fungal flora in both households and hospitals. Four *Aspergillus* species in total were cultured. *Aspergillus terreus* was detected in the hallway between isolation boxes and a nurse break room. *Aspergillus sydowii* and *Aspergillus versicolor* were cultured in samples from the personnel locks. Literature describes cases of onychomycosis [14]. These findings could be related to the personnel’s clogs, which are removed here. The three strains of *Aspergillus* detected in the cleanroom air of the personnel locks pose a potential risk for environmental contamination of the Transplant Unit. Despite all the advances in diagnostics and prophylactic antifungal therapy, invasive fungal infections have been on the rise over the last three decades. The upward trend of these infections is related to the growing population of high-risk patients and the increasing frequency of antifungal drug resistance [15].

Studies carried out in the 1970s and 1980s did not attach great importance to contaminated surfaces; today however these are viewed as crucial in relation to the possible spread of hospital-acquired strains. These are mainly bacterial strains of vancomycin-resistant enterococci, methicillin-resistant *Staphylococcus aureus*, *Pseudomonas aeruginosa*, and some viruses that in connection with the clinical course of the disease may contaminate surfaces quite far away from the patient, and subsequently spread through the hands of the personnel [16]. For immunosuppressed patients, all the Gram-negative bacterial strains we isolated pose a risk of infections with potentially severe to fatal consequences [17]. The personnel play an absolutely crucial role in the transmission of infectious agents in health care centres. However, staff cannot be eliminated from patient care. Their hands continue to be considered one of the most important vectors for transmitting infectious agents. Throughout the study, the personnel were monitored at regular monthly intervals. The most severe isolates were those from the hands (direct patient contact) and the nostril/nasal mucosa (carrier; particularly *Staphylococcus aureus*).

The hands of personnel have long been identified as a critical vector for the transmission of infectious agents and the emergence of nosocomial infections, as documented in recent epidemiological studies. In Sweden, Hedin and his team conducted a study that established that the hands of patients are an equally important vector for the transfer of infectious agents [18]. Most commonly, infectious agents are transmitted through contact between nurses and the patient.

## 5. Conclusions

An epidemiological study was held for the duration of one year at the Transplant Unit—Hemato-Oncology Clinic, University Hospital Olomouc. The study monitored microbial contamination of the cleanroom air, surfaces, water, colonization of the personnel by bacterial strains of epidemiological consequence, and found no significant deviations in the microbial contamination of the cleanroom air. The personnel entrance of the Transplant Unit represented a high risk area, as an extreme value (7270 CFU/m^3^) was recorded. Regime measures are fully effective, no other deficiencies were found. 
